# A Fatal Extrapulmonary Manifestation of COVID-19

**DOI:** 10.7759/cureus.14068

**Published:** 2021-03-23

**Authors:** Chad L Harris, Supraja Achuthanandan, Kiran Para, Vijay Shetty

**Affiliations:** 1 Internal Medicine, Maimonides Medical Center, Brooklyn, USA

**Keywords:** covid-19, spontaneous intracerebral hemorrhage

## Abstract

Coronavirus disease 2019 (COVID-19) grew to pandemic proportions in 2020. Research has shown that the causative virus, the severe acute respiratory syndrome coronavirus 2 (SARS-CoV-2), uses the angiotensin-converting enzyme II (ACE-II) receptor to attack host cells. These ACE-II receptors are present essentially in all organs, acting as a route of entry for SARS-CoV-2 to cause a wide variety of manifestations. There is growing research showing the neurologic effects of COVID-19. There have been several cases of encephalopathy, stroke, and encephalitis associated with COVID-19, however, intraventricular hemorrhages (IVH) have rarely been reported. Here we present a case of an IVH in the setting of COVID-19. A 32-year-old male with no past medical history, and not taking any medications, presented to the emergency room after acute onset loss of consciousness. Inflammatory markers were elevated, and computerized tomographic (CT) of the head and chest showed an intraventricular hemorrhage and bilateral interstitial infiltrates, respectively. Although possibly coincidental, this may represent a rare extrapulmonary fatal manifestation of COVID-19. With the growing evidence of neurologic presentations in patients with COVID-19, clinicians should maintain a high index of suspicion for COVID-19 to cause fatal extrapulmonary manifestations.

## Introduction

Severe acute respiratory syndrome coronavirus 2 (SARS-CoV-2) is a rapidly evolving respiratory virus causing mostly pulmonary manifestations with some reports of gastrointestinal, renal, and cardiac involvement. Neurologic manifestations of Coronavirus disease 2019 (COVID-19) are less common but have been observed in 36.4% of patients with severe disease, with 5.7% of these patients having acute cerebrovascular disease in a case series of 214 patients in China [1].

There has been emerging evidence of COVID-19 being associated with a myriad of intracerebral (IC) pathology such as stroke, encephalitis, and encephalopathy [2,3]. Cezar-Junior et al. [4] demonstrated four rare cases of subarachnoid hemorrhage in COVID-19 patients. Intraventricular hemorrhage (IVH) commonly occurs as complication of IC or subarachnoid hemorrhages; primary IVH are a rare occurrence, accounting for about 3.1% of all IC hemorrhages [5]. Other causes of IVH include but are not limited to vascular malformations, intraventricular tumors, intraventricular aneurysms, and coagulopathies [5-7]. In COVID-19 patients, primary IVH have not been observed.

COVID-19-associated pro-thrombotic state and coagulopathy are well described and have been shown to cause thrombotic events such as venous thromboembolism (VTE) and strokes [8,9]. IVH have been reported in a few critically ill patients with the use of anticoagulation [10]. We report a case of a young man who was found to have a fatal IVH with CT evidence and clinical manifestations of COVID-19, who was not on any antiplatelet or anticoagulant therapy.

## Case presentation

A 32-year-old man with a history of asthma, not on any prescription medications, presented to the hospital after cardiac arrest at home. Per his wife, the patient was in his usual state of health and then had sudden onset of blurry vision followed by dyspnea causing him to collapse. Emergency medical service (EMS) described the patient to be unresponsive with agonal breaths and frothing from the mouth. The patient was initially placed on a non-rebreather mask, subsequently switched to bag-valve-mask as he became hypoxic with bradypnea. Shortly after, he was found to be pulseless, requiring resuscitation and mechanical ventilation. He arrived at the emergency department in an irreversible coma.

**Figure 1:**
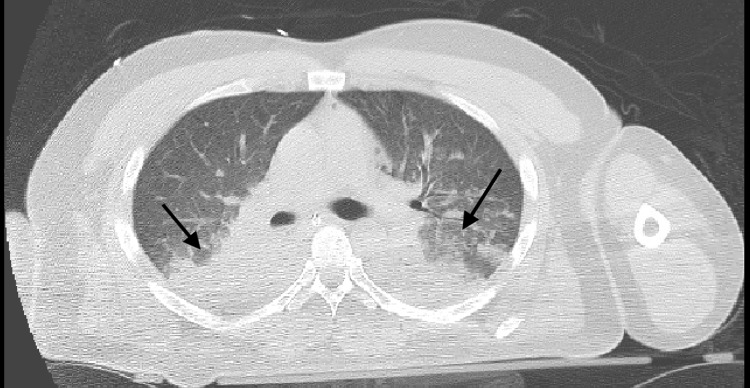
Computed tomography imaging of the chest without contrast demonstrating bilateral pneumonia highly suspicious of COVID-19.

**Figure 2:**
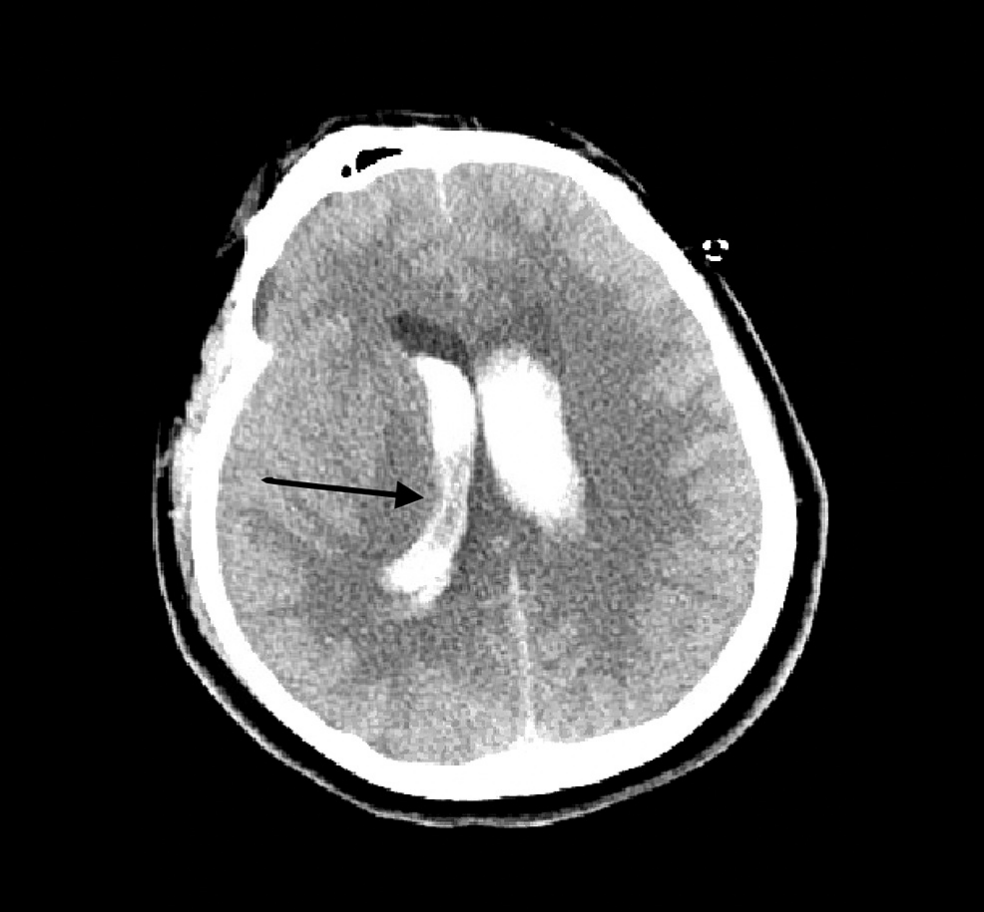
Computed tomography imaging of the brain demonstrating diffuse intraventricular hemorrhage with associated hydrocephalus.

The patient was afebrile with blood pressure 136/80 and tachycardia to 120. Electrocardiogram (EKG) at the time revealed sinus tachycardia. Neurologic exam was significant for fixed non-reactive pupils, absent brain stem reflexes with bilateral flaccid extremities. Glasgow Coma Scale at that time was 3. Initial labs revealed an anion gap metabolic acidosis (pH of 6.94 with AG, anion gap, of 24) likely secondary to lactic acidosis (lactate elevated to 14 mmol/L) and respiratory acidosis (pCO2 of 82 mm/Hg). There was hypokalemia of 2.8 mmol/L and mildly elevated creatinine of 1.3 mg/dL. Troponin was elevated at 0.79 ng/mL. Coagulation profile was within normal limits. Additionally, he was found to have leukocytosis of 15,800/UL with bandemia of 11%. He had elevated ferritin to 157 ng/ml and elevated lactate dehydrogenase (LDH) at 315 IU/L. Urine toxicology revealed positive for marijuana but not for opiates or benzodiazepines. Chest x-ray revealed bilateral pulmonary infiltrates. CT imaging of the chest without contrast showed bilateral pneumonia highly suspicious of COVID-19 (Figure 1). CT imaging of the brain revealed diffuse IVH with associated hydrocephalus and no obvious parenchymal source of bleeding (Figure 2).

The critical care, stroke, and neurosurgery services were all consulted. National Institutes of Health Stroke Scale (NIHSS) was calculated to be 36, consistent with 100% mortality rate; hence, no acute intervention was recommended. After an extensive discussion with family regarding patient’s prognosis, the patient was palliatively extubated and expired. A COVID-19 test was not obtained. The family did not wish to pursue an autopsy.

## Discussion

A number of IC manifestations of COVID-19 have been reported, mainly stroke, encephalitis, and encephalopathies [2,3]. Very few IC hemorrhages have been reported, most of which were found to be subarachnoid hemorrhages [4]. This case is of a young man with a fatal IVH without any obvious source of intraparenchymal hemorrhage. Recent evidence shows that severe COVID-19 infection can be complicated with coagulopathy, which presents with pro-thrombotic events leading to venous thromboembolism, pulmonary embolism, and strokes; hence, the use of anticoagulation in the COVID-19 population has become unanimous [11,12]. However, hemorrhagic manifestations in the setting of COVID-19 have not been fully understood. This young male was not on any anticoagulation or antiplatelet medication at home and had no significant comorbidities. Although a COVID PCR test on this patient was not obtained, clinical, laboratory, and radiologic evidence all suggested COVID-19.

The mechanism of COVID-19-induced IVH is not fully understood. SARS-CoV-2 binds to the angiotensin-converting enzyme II (ACE-II) receptor for host cell entry, much like the SARS-CoV-2 and Middle East respiratory syndrome (MERS-Co-V) viruses [9,13-15]. ACE-II receptors and angiotensin II receptors are widely expressed in both the lung type 2 alveolar cells and the epithelial cells of the gastrointestinal (GI) tract, two systems where COVID-19 is known to have symptoms. ACE-II and angiotensin II receptors are also highly expressed in the brain, specifically in the cerebrovascular endothelial cells [16]. These cells play a key role in vascular autoregulation and cerebral perfusion [17]. Therefore, SARS-CoV-2 would theoretically bind to the cerebrovascular endothelial cells causing neurologic pathology. Additionally, autopsies done on COVID-19 patients have found evidence of systemic vasculitis and more specifically, vasculitis of cerebral venules [18]. Studies have also shown that the virus can be found in the CSF [19]. Hence, it has been hypothesized that disruption of cerebral autoregulation caused by dysfunction of brain ACE-II receptors as a result of SARS-CoV-2 would lead to endothelial damage and rupture of arterial wall, causing hemorrhage [13]. However, further research is needed to understand the exact mechanism of COVID-19 causing IC and IVH.

## Conclusions

IC hemorrhage as an extrapulmonary manifestation of SARS-CoV-2 is rare and can be fatal. Current literature does not have enough evidence to prove or hypothesize the hemorrhagic and extrapulmonary manifestations except for their entry through the ACE-II receptors. More studies are required to understand detailed mechanisms for extrapulmonary manifestations of COVID-19.
